# Medical Relief of the Sick Poor in Rome and Prussia

**Published:** 1840

**Authors:** 


### MEDICAL RELIEF OF THE SICK POOR IN ROME AND PRUSSIA.

I. Reminiscences of Rome: or, a Religious, Moral, and Literary View of the Eternal City, in a Series of Letters addressed to a Friend in England, by a Member of the Arcadian Academy. London, 1838, pp. 298.

II. Reglement fur die Stadt-Armen Krankenpflege der Hiesigen Commun, &c. Berlin, 1823.

Rules for taking care of the Town's Sick-poor, &c.

III. Summerische Uebericht der im Jahre 1838, im Königlichen. Charite-Krankenhause verpflegten Kranken, nebst eivem Ueberblick der Beranderungen dieser Austalt in dem letzten Decemnium. Berlin, 1839.

A Summary Account of the Patients who were treated in the Charity Infirmary during the Year 1838; and a Statement of the Alterations which have taken place in that Establishment in the course of the last ten years.

In the present article it will be our endeavour to present a complete view of the provisions for the relief of the sick-poor, in the principal capital of Christian Europe, " where every stone is a book—every inscription a lesson —and every ante-chamber an academy ;" and in the chief kingdom of Protestant Germany, the paternal character of whose king, and the mild despotism of whose government, appear to have been mainly exercised in improving the social condition of the people at large.

Travellers and tourists even of the medical profession, when they visit Rome, visit her as " the ancient emporium of art and science," and view her only under the appearance of " a time-worn and decrepit matron sitting disconsolate amid the tombs of her departed heroes—her sages—and her saints." And when they visit Germany, it would seem as though they did so merely to reverberate the echoes of other men's admiration and wonder, who had gone before, and already surveyed the grandeur of her hills, and the magnificence of her rivers.

Heroes, and sages, and saints, even in this the 19th century, when intellect is said to be " on the march," are still exceptions to the average stature of mankind considered in relation to the prowess, the wisdom, and the goodness of the mass of working, walking, and talking human beings. Of Howards and Frys we are only permitted to see one in an age. And to this cause it is perhaps owing that we may search through more than a hundred volumes of tours on the Continent, and travels in Italy and Germany, without being able to glean any satisfactory particulars respecting the poor of those countries, and especially the sick-poor.

From a Roman Catholic priest in our neighbourhood we have received, in the volume which stands at the head of this article, a pleasing exception to the general rule, as respects the "Eternal City;" while a Protestant gentleman of the legal profession has transmitted us the regulations for taking care of the town's sick-poor, and the excellent report by Rust of the Royal Infirmary of the Charité at Berlin. And by means of these we proceed to make our readers acquainted with a feature in the civil aspect of two very differently constituted States, with which travellers and tourists in general have failed to render either the public or themselves familiar. And, we choose the present time for doing so, on account of the pending discussion in Parliament of the great question of Medical Relief to the Sick Poor—a question which will only then be settled when the basis of any provision for rendering that relief satisfactory to the medical profession and to the poor, shall be one of justice to the former—and mercy to the latter.

I. We begin with Rome. One of the officers of the Papal household is called "l'Avvocato de Poveri." The almoner, generally an archbishop (in partibus) is another officer at the Pontifical Court. The chief business of the former is to watch over the interests of the imprisoned—of the latter, to provide for the necessities of the indigent poor.

Medical assistance and medicaments are gratuitously provided out of the Papal Almonry, "for bashful paupers, who are ashamed to apply to the public hospitals for relief. The almoner, who keeps a list of this class of persons, appoints deputies to see them properly attended at their own homes." And, .or the better distribution of the relief thus provided, eleven clergymen, eleven physicians, eleven surgeons, eleven chemists, and two druggists are selected, and employed in the different wards of the city.

It is calculated that, in almonry, the Pope bestows annually not less than 50,000 crowns; which sum, though considerable, does not amount to the half of what his almoner had to dispose of prior to the French Revolution. "The other public institutions of charity in Rome, have also revenues to the amount of 764,000 crowns a year!"—*Reminiscences of Rome, p.* 179.

The *public hospitals*, considering the comparatively small population of the city, estimated at about 160,000 inhabitants, are indeed magnificent, but their magnificence, like the dome of St. Peter contrasted with the surrounding buildings, is utterly disproportionate to the extent of the city, or its populousness. For example, the Hospital of San Spirito, for males alone, is capable of containing 3,000 beds; and its annual average of inmates is about 12,000. The hospital for females, again, Archiospedale del Santissimo Salvatore, contains 600 beds, and admits annually about 3000 patients. The surgical hospital, for the reception exclusively of accidents, receives 900 patients annually. The hospital of incurables contains 350 beds; and the annual average of patients is not less than 2000. The number of convalescents annually received into the hospital of the Holy Trinity, from other institutions, for the furtherance of their recovery, amounts to 15,000. The hospital for cutaneous diseases contains 200 beds, and receives upon an average 400 patients in the year. The fever hospital has 80 beds, the number of its annual inmates is not stated, although, from so fruitful a source as the Pontine Marshes, we may be sure it is constantly filled; and from the limited duration of most cases of fever, we may be also certain that its tenants in the course of the year will be altogether out of proportion to the size of its dormitories, or the number of its beds. The lying-in hospital, during ten years, has received nearly 2000 pregnant women. And, in the Roman lunatic asylum, there are accommodations for 400 patients, and the number of admissions annually amounts to 100.

Taking the sum of the foregoing particulars, it will be seen that, in Rome, with a population of 160,000, and without enumerating 18 minor hospitals for poor sick foreigners, there is hospital accommodation for the sick, the hurt, and the scabbed, the convalescent, the incurable, the lying-in, and the lunatic, to the amount of not less than 5000 beds; and, there are actually accommodated upwards of 34,000 patients. This exhibits certainly a frightful extent of bodily suffering in so small a community, but displays also, in favourable contrast, an immense expenditure of human beneficence to alleviate that suffering.

The proportionate mortality in the various institutions differs very greatly; Morichini (degl'Instituti di Carità) calculated the number of inmates in the Hospital of St. Spirito at 12,000, and the mortality at from seven to twenty per cent. "During the year 1832, according to the official register, of 15,524 patients admitted, only 1246 died in the hospital!"—*Reminiscences of Rome, p.* 183.

In the hospital del Santissimo Salvatore, 12 per cent. is the average ratio of mortality. In the surgical hospital, the member of the Arcadian Academy tells us that "not more than five per cent." of the patients annually admitted, "succumb under treatment"—which expression, we presume, implies, that this is the proportion of cases which prove fatal.

In the Hospital of St. Giacomo, for incurable invalids, about 200, or 10 per cent., die annually.

In the Hospital of St. Gallicano, for diseases of the skin, 30 deaths out of 400 patients is the calculation.

We have no data before us from which to fix the ratio of mortality in the hospital cases of fever.

Of 2000 lying-in women, delivered in the St. Rocco Hospital, not more than 12 died in childbed, although, according to Morichini's calculations, instrumental assistance was employed in the ratio of five per cent. of the whole number of deliveries.

A third part of the insane are reported to recover—the number that die are not stated.

The *medical administration* of the hospitals above enumerated may be understood from an attentive consideration of the following particulars:— The Hospital of San Spirito has twelve physicians, six of whom are assistants or "sostituti" only, and four surgeons, with 100 resident servants. To the hospital "del Santissimo Salvatore," four physicians and three surgeons, with assistants, are attached—"the ordinary operations of surgery, such as bleeding, &c. are performed by the resident nuns themselves." 190.

To the surgical hospital there are four surgeons and two physicians. To the hospital for cutaneous diseases, three physicians and three surgeons, with about 40 other attendants. The hospital for incurable invalids has four physicians and four surgeons. With the exception of one extra physician, the entire service of the hospital for fever patients is performed by the confraternity or religious order, known as the Fate Ben Fratelli, or Do-good Brethren, "who attend the sick confided to their care night and day, ministering to their spiritual and temporal wants with the utmost solicitude and attention." 197.

The *revenues and œconomic management* of the foregoing institutions are confided for the most part to the clergy, and are generally ample. The revenues of St. Spirito considerably exceed 100,000 crowns per annum—it was given in charge by Pope Innocent III. to a religious order of Hospitallers, founded in France, by Guy de Montpellier, as the order of the Holy Ghost. The revenues of the Hospital del Santissimo Salvatore amount to 32,000 crowns a year, and are administered by a lay and ecclesiastical committee, with one of the cardinals for president. Those of S. Maria della Consolazione, (for accidents,) and amounting to 12,000 crowns, are managed by a special committee, appointed by the Papal Government. A committee, composed of two ecclesiastical dignitaries and a lay gentleman appointed by the Pope, act as trustees for the administration of the revenues (of the Hospital of St. Giacomo) amounting to about 30,000 crowns per annum. The Trinity, or Convalescent and Pilgrim Hospital, is endowed with property to the value of 18,000 crowns a year—and the Government contributes to the hospital for the maintenance of each invalid soldier, fourteen bajocchi, or halfpence, per diem. The funds are managed by trustees, appointed by the arch-confraternity, "Della S. S. Trinità de Pellegrini, e Convalescenti." The lying-in hospital was endowed by Cardinal Salviati, with a revenue of 2,500 crowns per annum. The lunatic asylum has an annual revenue of 12,000 crowns, two-thirds of which are furnished by the State. The Pope's exchequer also defrays the expences, 12,000 crowns per annum, of the institution for patients with cutaneous disorders. His chancellor appoints the committee of management in this instance. The lunatic asylum is superintended by a lay and ecclesiastical committee, under the presidentship of the Commendatore di S. Spirito—a prelate of the first rank in the Roman Court. The gross revenue of the whole, not including that of the fever hospital, amounts to 228,500 crowns per annum.

It is no part of our business as medical reviewers to meddle with the religious opinions of men, or to discuss different modes of faith—but we may observe here, that, to the predominance of religious habits and feelings in the Roman community may be traced one of the most pleasing, if not the most pleasing feature of their system of administering medical relief to the sick poor, we mean the substitution of voluntary for hired nurses, and the very ample, and, to our Protestant eyes, excessive provision for their spiritual wants.

In the large hospital for females, and in the female department of that for incurables, a community of female hospitallers, introduced from Genoa in imitation of the French Sisters of Charity, attend to the sick as nurses, and themselves bleed and perform all the minor operations of surgery. In that for incurables, St. Camillus, in 1584, founded his order, the members of which have the spiritual direction of it still. This order, distinguished from the other clergy by a red cross worn on the upper part of their black mantles, and known as the "Chierici, Regolari Ministri degli' Infermi," "are obliged by vow to attend upon the sick at their own homes, when sent for, even during plague, or any other contagious disease."

The reflections of the author of Reminiscences of Rome upon this part of our subject, although not new, are nevertheless true, and being true, deserve the serious consideration of those to whom is confided the solemn duty of legislating for the more effectual administration of medical and other relief to the sick poor of our own country. We entirely agree with him that—
" It would be highly advantageous to the poor in our own, and, in fact, in every country, if hospitals were generally confided to the care of such administrators and attendants as the 'Fate Ben Fratelli' in Italy, or, as the 'Sœurs de la Charité' in France, who certainly make the best nurses, combining, as they do, more intelligence and a higher sense of duty than is usually found among uneducated and hired servants. It is not merely the nauseous medicine doled out from the dispensary, or the scanty pittance of weak broths and coarse meats, served up with the harsh accompaniments and cold formality of English parochial and workhouse discipline, which the sick or the dying patients require from their attendants; for the kindly look and devout consolation, excited by a feeling of religious sympathy, are more refreshing to the aridity of the languid frame, than a cooling draught is to the parched tongue."

There is yet one thing more which we must notice, and notice with commendation, at the same time that we express our astonishment at the total absence of every thing like it in the capitals of our own country. We refer to the subservience of the medical institutions of Rome to the improvement of medical students, by the existence of numerous scholarships.

About fifty medical and surgical students are boarded and lodged in the Hospital of San Spirito. In that of S. Maria della Consolazione, ten surgical students are so boarded and lodged. Fifteen medical students are maintained on the establishment of St. Giacomo. Throughout the United Kingdom of Great Britain and Ireland there is no such provision for deserving students. The wealthy, indeed, may become residents in scant numbers, but only upon payment of a sum adequate to their board and lodging elsewhere.

Before taking our leave of the Ancient City, we would mention a curious custom which still holds there; and place on permanent record a fact stated by the anonymous author of the Reminiscences, which, if true, deserves to be not only carefully cherished but widely circulated, as an anecdote of excellence in high places, not the less creditable to the party concerned, because a sovereign pontiff.

The bodies of those who die in San Spirito, " are not removed from their beds until two hours after death has ensued. They are then transported to the Mortuary Hall, where they are kept, previous to interment, for twenty-four hours. Every evening, as soon as the Ave-Maria bell rings, a charitable confraternity of laymen come processionally with lights, and a crucifix, to convey the deceased to the burial-ground, situate on the Taniculum. When it happens, as is frequently the case, that there are no dead to be interred, these pious penitents nevertheless, even in cold and stormy weather, make their usual pilgrimage to the hospital cemetery, reciting prayers for the faithful departed."

" Abuses, nevertheless, will sometimes creep into the best regulated establishments. A remarkable instance occurred to my recollection, in this vast hospital, under the pontificate of Leo XII. This pontiff, so noted for his vigilance over the conduct of his ministers, in every department of public administration, used to inquire into every disorder, and listen to every complaint. Suspecting that all was not right at San Spirito, he, followed by a few confidential attendants in disguise, unexpectedly made his appearance at the hospital, about two hours after midnight, on the 25th of June, 1825. The Pope, while hastily examining the different wards, perceived that one of the poor patients was nearly at the point of death, without a single resident clergyman being in attendance, as in duty bound, at all hours of the day and night, to administer the sacraments to the dying. He, in consequence, immediately dispatched one of his own chaplains for the viaticum, and in the meantime placed himself alongside the dying man, to hear his confession, and to impart to him the consolations of religion. Ere long, the news of so unexpected a visitor's arrival had spread with the rapidity of lightning; and you may imagine, better than I can describe, the stir it made among the healthy and slumbering guardians of the hospital. The zealous pontiff, however, did not desist from his pious undertaking until he had comforted the departing soul with the bread of eternal life. He then quitted this receptacle of infirmities, accompanied by the prayers and blessings of the helpless patients, and the trepidation and astonishment of those whose negligence he had thus so flagrantly though tacitly rebuked."

"A medal was struck, (though not by the hospitallers,) as a memorial of this event, and a picture, representing the pope's nocturnal visit, may be seen in the Altieri Palace. The present nunzio at the court of Vienna was one of Leo 12th's attendants on the occasion."—*Reminiscences of Rome,* pp. 183-4-5.

II. Turning to the Medical Institutions for the relief of the Sick Poor in Prussia, we observe an inverted order of things. Instead of the State being there subservient to the Church, the Church in almost all things is rendered subservient to the State. And, accordingly, we may expect to find the glory of its institutions of mercy gathering round the civil, rather than the ecclesiastical establishments.

In Prussia, it is the recognized obligation of every parish to provide for its own poor, so far as its abilities and their wants can be said to correspond. Medical relief for the sick poor is implied in such provision, which devolves as a duty upon every commune, and is shared, in particular cases, by the Government, which represents the whole country.

There is, however, no general organization, of a separate and distinct character, for providing such relief, which can be termed national; or which is of uniform applicability throughout the country. Hence, while such relief is indeed afforded everywhere, it almost everywhere presents a varied and varying aspect according to the differences of locality, population, &c. And thus, while in cities the highest order of medical officers, physicians for the poor, is always to be met with; in most of the rural districts they are extremely rare; and only generally to be found in those of the richer, or Rhine provinces.

The medical system, as a whole, is subject to the Minister of Health, who, for convenience, is also minister of education, and ecclesiastical matters. He is assisted by two medical councils, one for contrivance, the other for execution; to either of which he applies according to the nature of the case. Under him are *all* medical men, and he exercises his control by each provincial government, who again employ functionaries to carry it out in the respective districts, by a general superintendence of medical matters. If the poor were to be neglected by the commune, it would be reminded of its duty, in Berlin—by the police; in the country—by the provincial government.

No medical man can practise at all in Prussia without a government approbation in addition to a medical degree—and if a similar rule held in our own country, it would at once remove all the heart-burnings which vex and irritate the profession from one end of the United Kingdom to the other. Of course no medical man can fill the office of a poor's physician or surgeon without the double qualification.

In the Royal Domains, the fiscus lies under the same obligation to provide for the poor generally, and for the sick poor in particular, as any other proprietor. And such institutions as were of old endowed by the state, or serve a double purpose (as instruction,) are maintained by Government.

So far as the people at large are concerned, medical relief is, as has been already stated, more generally left to be provided according to the discretion of particular communes, than ensured by any governmental or national provision.

In the larger communes, accordingly, there are infirmaries, into which those only are received who cannot have proper nursing and attendance from their own families, or at their own lodgings. In the smaller, and especially the country, parishes, the authorities contrive to do without infirmaries, or contract for the maintenance of their sick at the adjacent city one. In some districts, again, the parishes, when too feeble, singly, to undertake the cost of such establishments, have united for the erection of district lazarettoes. When they possess the means—which, however, is rarely the case, except in the Rhine provinces, and in large cities, they are at liberty, but not under obligation, to appoint physicians for the poor.

In the principal cities, especially Berlin, there is, as a civic institution, a public and organized care of the sick poor. Here, also, associations of private individuals are in more or less active operation; but these are only indirectly or not at all connected with the public institution. The latter meets what law and necessity require, the former prepares a way for benevolence. In Berlin there is a poor's-rate for the sick, as well as for the healthy poor, and any inadequacy of this public fund to meet exigencies of an extraordinary kind is met by the liberality of voluntary contributors.

This public institution is known as "the Poor Direction," a board composed of professional men and others, and consisting, in Berlin, of the lord provost, the members of the magistracy, an equal number of town deputies elected by the citizens, and a number of physicians, many of whom belong to the Medical College. Where the field of their operations is divided, each division, or district, is subjected to a commission, or sub-direction. In Berlin, under the central board, there are not less than fifty-eight such commissions, each of which constitutes a small college with a president or manager, to whom the pauper has recourse, and who refers him to a doctor, if in need of one, with a written recommendation. In other cases, the pauper, when sick, sends for the "poor deputy," who ascertains whether the patient be able to pay for medical attendance and medicine, or not:—if not, he gives him a certificate to the poor physician or surgeon of the district, who is expected to see him as soon as possible. Except in cases of extremity, when he is competent to act alone—the deputy is assisted by the presence of a member of the commission; from which a report of each case is to be sent to the central board.

The recognized objects of gratuitous medical relief are—the poor. And, in the eye of the law, *he* is poor who possesses insufficient means, or inadequate strength to provide himself, and those belonging to him who are incapable of earning their own subsistence, with the mere necessaries of life, as food—clothing—shelter, and fuel. He who is not poor in this sense, has no claim on the public for support, and belongs—if not to the rich—to that class of persons which is supposed to be beyond the pale of the poor-direction. When a man, legally poor, falls sick, the poor's physician of the district decides upon his case, and whether indeed he be sick at all or not; and, upon the judgment of the physician, the disposition of the higher administration, and the local and other family relations, the amount of the help to be afforded him depends. And thus the relations of the sick poor to the rest of the community regulate themselves, rather in a practical, that is, administrative, than in a theoretical or legal way. While every sick person for whose case the commissioner has decided relief to be necessary, is gratuitously attended and relieved accordingly, and while the circumstances enumerated above, assign the only limits to that relief; a commune is at liberty, if it sees fit, to go beyond the bounds of simple objection—there being no law to prohibit its passing out of the province of police into that of benevolence. The boundaries of medico-parochial relief are thus rendered difficult of discovery, and can only be rightly observed by an arbitriment not too profuse, in order that private benevolence intrude not too much into the poor's administration; else those who are without means, yet not reckoned as destitute poor, might be pampered and encouraged to demands which would exhaust the poor's funds, and involve the commune in inextricable difficulty. A drunkard, a spendthrift, or one who does not receive the medical officers with due respect, loses his right to medical aid, if his reform be hopeless.

The pauper lunatics are received into Provincial Asylums for cure, where cure is practicable; for custody and protection, when incurable; these asylums are under the sanction of the estates of the province, and are helped to be maintained by pecuniary assistance from Government. In most of the provinces there are institutions of the above description—and measures are in progress for supplying them to all. In the New Charité at Berlin there is an Insane Department. The Lunatic Asylum at Leyburg is the largest provincial one.

Poor lying-in women are provided with midwives, gratuitously, in every district. These district midwives are bound to the care of the poor, but not exclusively. They are selected from the midwives who have been educated at institutions for the purpose, partly supported by Government, and partly at the expense of the provinces, and undergo a strict examination. In the very smallest village there is always a midwife, who is obliged to render gratuitous aid, if necessary, to any poor woman unexpectedly taken in labour. But an application for such help should be sent in, in time, to the local authority, (in Berlin to the poor's commission,) which then provides a midwife in readiness—who is not, however, permitted to undertake any but natural labours; for where manual help is required, an accoucheur must be called in, who acts, in general, gratuitously, but only so at his own option. The physicians and surgeons are, generally, accoucheurs as well, and, therefore, can act as such, in the cases of the poor—but, in order to practise as accoucheurs it is necessary, in their cases, as well as in that of the mere midwives, that they should previously undergo an especial examination in obstetricy.

In the Berlin Charitè every applicant in her ninth month of pregnancy is received impromptu in order to her confinement: and so, likewise, in the Royal Lying-in Institution attached to the University, if she be poor. In the provinces it is otherwise, no fixed rule obtaining, but the principle upon which all regulations are based, being, that the local authorities or commune must see that the pregnant as well as the sick and other poor are provided for. Midwives act commonly, but the parturient woman who has laid claim to public help, cannot decline male assistance. In the hospitals, on the contrary, the accouchements are either conducted solely by accoucheurs, or superintended by a professor of obstetricy when conducted by students learning midwifery, whether male or female. In Bönn, the poor administration pays medical men and midwives to attend: the midwives are all well-educated, and duly examined.

When the situation and circumstances of a sick or lying-in pauper are such as to require the presence of an attendant or nurse, the poor commission provides this also. But this is the exception—the rule being, that the sick and lying-in poor shall be attended and nursed by their own relatives; or, when solitary individuals, that they go into a hospital. The prejudice against hospitals of every kind is fast decreasing. Where nurses are provided at the public expense, they are not remunerated by a fixed salary, but according to place, work, and requisite qualifications. When the patients are transferred to hospitals, if unable to walk, they are conveyed thither free of expence, in a carriage kept for the purpose in every district; and for cases which might suffer by the motion, even of a carriage, sedan chairs and litters are provided.

The sick poor of Berlin have access to Russian baths, sulphur baths, and baths of different kinds, with herbs, &c. and even have them brought to their rooms when unable to leave them. When recommended by the poor's physician for removal to the Gailbrunn (watering place) they are sent there at the cost of the poor's administration, and provided with free board and lodging.

The necessary medicines are not furnished either by the government or the medical officer ; but by the apothecaries, who, alone, have this privilege. The Prussian apothecary, however, can give no medical advice, but, nevertheless, his education is obliged to be of that thoroughly practical kind as to enable him to compound all the preparations he may be called upon to dispense. On the other hand, the physician and surgeon, whose prescriptions he is bound to " observe, execute, and preserve," as his vouchers for his termly charges to the Poor Commisson, dare not dispense the medicines they prescribe. The quarterly accounts of the apothecary, proved by the receipts of the Poor's Physicians and Surgeons, are, after regular revision, according to the "medicine taxation," honored by the treasurer of the Poor's Direction.

Instruments, bandages, &c. are granted by the central board, on the attestation of the surgeon that they are needed—on receiving which attestation the " Bandagist" gives them out. Beds, cloaks, shirts, flannels, are in like manner at the command of the medical attendants for the use of their sick; and when so used by them a quarterly account is rendered to the Board.

In Berlin, it is the duty, moreover, of the respective Poor Commissions to carry out all recommendations of the medical officers, for facilitating the recovery of the sick poor; and, on their report, everything comprised under the term "medical comforts" is unsparingly afforded. A restaurateur supplying the prescribed dietetic necessaries, such as—beer, beef, soups, eggs, brandy, coffee, wine, &c. upon the presentation of tickets signed by the medical attendant.

In cases where the sick pauper is the head of a family, or its chief support, the wants of that family are attended to by the Poor Commission of the district within which it resides, which must take cognizance of the case, and either grant the family sufficient support at home, or remove its members into hospitals, workhouses, orphan houses, or the like, as they may judge expedient. This is generally done on a report to the Commission by the physician ; and the allowance, if in money, is proportioned to the funds placed at the disposal of the Commission.

All the principal cities, as has been stated before, have infirmaries. These severally rest for support, partly on institutions—partly on the means of the communes—and partly on collections. They are distinct from the royal infirmaries, in not being government establishments. In Berlin, "the Charitè" is a mixed institution. State and commune, both, contribute to its maintenance; the State, for this reason, that it is also an institution for instruction. As we shall notice it at large, presently, we postpone for the present, a further description of it. The academy of military medicine, (La Pepiniere) is connected with the university, in which latter every military physician must graduate as a doctor of medicine and surgery, none others being admitted to the medical charge of any government hospital or infirmary. There are besides, in Berlin, several hospitals for the aged and for incurables. The Jews, and the French colonists, have separate hospitals of their own for occasional disease. In the Provinces and Provincial towns the hospitals are well kept and ably managed—but other than military men are admitted to a share in the medical arrangements. Of these, the government pays the expences, out of a tax levied for the purpose, while it encourages and calls for voluntary contributions. Where the city infirmaries are supported out of the commune funds, the commune appoints the medical man. Where there are only royal infirmaries, the commune possesses the limited privilege only, of having a certain number of its poor as inmates, free of charge—when they require to exceed the number so privileged, they must contribute according to a fixed rate for every additional pauper from their districts—for example, at the Royal Charitè, in Berlin, the commune is entitled to 100,000 days' food per annum, for their sick, 100 days food that is, gratis, for 1,000 sick, to be distributed among what number they please. All beyond that amount is charged against them. For the hospitals in general there are no universally applicable normal regulations ; particular infirmaries have their particular rules, and the laws of administration proceed from the instructions of the officers. The administration of all Prussian hospitals is under the care of government.

The extent of the medical districts, and the amount of population assigned to the medical officers individually, cannot be ascertained with exactness, because each commune alone knows the necessities of its own population, and the extent to which it will go in providing for their relief. Of 272,000 inhabitants, the population of Berlin, 20,000 come under each of the district physicians and surgeons ; but very small towns, of perhaps only 1 or 2,000 inhabitants, have a district physician or surgeon for their own poor alone.

It is thought to depend entirely on the locality. The introdnction of district physicians, into the provinces generally, has been, hitherto, found impracticable. In Berlin, where there are 20,000 inhabitants and upwards to one poor's physican and one poor's surgeon, the number of poor in such a population is very different in different districts. The number of districts here is twelve; and the number of people, and of poor especially, varies very much in each. But the average number of pauper patients daily attended in each, is estimated at 20. The arrangement in provincial towns is seldom anything else than a meagre imitation of that in the metropolis. In the country, each parish has a physician and surgeon, with a small remuneration from government, but the poverty of the people prevents the relief from being provided as in towns.

The duties of the medical officers take different forms in the different parishes. In small ones, mere formalities probably do not exist. In general, the contract made with the medical man is his instruction. In Berlin, the physicians and surgeons have their duties more exactly defined by precise instructions and regulations, which are too extended to introduce in this place. Every quarter, lists of the reception and departure of sick persons are given in to the Poor Direction. From these lists a combined view is framed and forwarded to the royal police residency, the health functionaries of which take cognizance of the same in a health police point of view.

The poor's physician is not merely responsible for visiting the poor, but is also an officer for the care of the general health of the community, and is therefore expected to direct the attention of the police to every evil by which the health of society at large may be affected. He has to institute medico-topographical surveys, in order to the prevention of contagious and other diseases, and can make the police punish dirtiness in the streets and houses, bad habits, &c. they being bound to follow his preventive directions. On receiving his appointment he has to take an oath for the faithful fulfilment of his duties.

In addition to the quarterly return already mentioned, he has to make out a monthly return as well, for the Poor Direction, in the following form :—

| Name. | Age. | Ab ode. | Rank or calling. | Disease. | How long ill when first seen. | Date when first visited. | Dismissed cured. | „ uncured. | „ dead. | „ to the hospital. | General Remarks. |
|---|---|---|---|---|---|---|---|---|---|---|---|
| | | | | | | | | | | | |

The medical man of the district to whose care the patient is committed, is expected to see him as early as possible after receiving notice of his illness, to treat the case himself, and if the illness be acute and dangerous, to visit him *at least once a day*. And, should it be a chronic or slight ailment only, to see him, at least, twice a week: in fatal cases the sworn medical man has to give a certificate of the death, its place, time, and cause. There is no inquest of any kind held upon the body of a deceased pauper.

Except the ordinary qualifications already stated for the legal exercise of the medical profession at all, there is nothing required in order to an appointment either by government or the commune, as poor's doctor, or medical officer for the care of the poor. The required examinations, however, are so extensive and so strict in Prussia, that Prussian certificates are often received as sufficient in other German states. The strictness varying according to the degree aimed at—that for a surgeon being less than that for a physician, the reverse of our English custom. But then, a physician is not a mere physician, but one appointed by government to take charge of medical jurisprudence, or medical police, or both. No one who is not a graduate of medicine and surgery in one of the five Prussian universities can become a physician. Hence no surgeon can become a physician without recommencing the prescribed course of study for a physician. And none is allowed even to enter upon the study of medicine who is not a bachelor of science, Anglice, master of arts. Most physicians are also operative surgeons, and, in general, the minor operations alone are performed by mere surgeons.

The only gradations of rank among the medical officers of the poor, are such as their professional, not their official, position, confers. For instance there are two orders of physicians. 1. Physicians and surgeons combined, in a word, general practitioners; these are promoted from—2, the mere physician, and acquire the title of *doctor* (med. doct.). 3rdly. There are two classes of surgeons—graduates in surgery and non-graduates. In infirmaries, according to their size, there are directing and assisting physicians, but these must all, in the royal infirmaries, have been *military* medical men.

The *appointment of medical officers* emanates from different sources, as the State, the Commune, or, in cities such as Berlin, the Poor's Direction: and are held upon various tenures, either, ad vitam aut culpam—during pleasure—or, with a reservation of six months' notice before relinquishing or being dismissed their offices.

*Promotions* are regulated by the minister of the Department.

They are subject to no other general oversight than all medical persons or other functionaries. If physicians or surgeons are in fault they are responsible to government, in which, as well as in the ministerium for medical affairs, higher medical functionaries are set to act for the king. The poor's physicians, &c. are also responsible to the party employing them, the Poor Direction or the Commune, as the case may be. But, although the particular administration has power to admonish him, he can only be dismissed after a formal judicial inquiry. And this is necessary to the safety of the individual, and the preservation of his character, for every practitioner is severely punished for mal-practice. In cases where a patient complains of injury sustained from his want of skill, mal-treatment, or neglect, the practitioner complained of is tried before the criminal court. In cases where the patient is poor, and complains of the poor's physician, the board under which the physician is acting is bound to investigate the case, and if the complaint be of a serious character, and duly established, he may be dismissed. If his offences be of a minor kind, yet such as to render the dissolution of the relation between him and the Poor's Direction desirable, he receives notice to resign. If of a graver kind, he undergoes the legal penalties, for which application is made to the criminal court.

They are very inadequately remunerated. Experience and distinction being the equivalents with them as with us for adequate salaries. In Berlin, the poor's physicians receive £30 a year each—the poor's surgeons £8, £10, and £15 per annum. In the country, the medical officers for attending to the sick poor, are variously paid, some receiving 50, some 100, some 200 dollars, (3s. English). The doctors of midwifery ask no fee for attending the pregnant poor, midwives get a small one, varying from one to five dollars, on producing a certificate from the poor's physician. In small towns this is only one or less.

Between the medical faculties in their corporate capacity, and the medical districts, there is no necessary connexion subsisting. When any such subsists it is only by voluntary agreement between the magistracy and the university, as in Bönn, where the poor are cared for by the clinicum, and the city pays a certain sum for medicines. The universities being connected with the care of the sick poor only in so far as *clinical institutions* are, for the sake of instruction, maintained by the State; and then, such patients are taken care of, either at those institutions, or at their own homes, by the ambulatory clinic, and gratuitously supplied with medicines, in aid of the Poor Commission. In the Royal Charitè at Berlin, there is this peculiarity :—the State must pay for the poor who go or are sent thither to be used for instruction, at the fixed rate of ¼ dollar daily.

Several of the districts in Berlin are connected with an ambulatory clinical school; and in them the poor's physician is an assistant physician to the school; and the sick poor, who apply to the poor's physician, are visited by the young medical men under his direction and that of the professor of clinical medicine. In Berlin, also, an ambulatory clinical school is united with the lying-in hospital; three assistant physicians directing the labours of the students or graduates through the city and suburbs, and the school providing nurses to attend the female during her first week, and assist her with her child. The lying-in-ward of the Charitè has a school for students and midwives for Bönn and Brandenburgh, under a director; and this is imitated in the provinces.

There is one other particular connected with the Prussian system, which is worthy of notice :—In cases of feigned sickness and pretended poverty, a medical officer discovering the fact announces it to the poor's direction, and the impostor is handed over to the criminal court to be punished at common law. Mere pretexts of sickness and poverty, however, when detected in time to prevent imposition, lead to no punishment. In both cases, it must, of course, be difficult to detect the actual facts, for the ideas men have of poverty are all relative. But, wherever positive fraud has been perpetrated, the perpetrator is punished as any other common criminal would be. The poor-direction too, can recover by civil process, for support granted on false pretences to parties, not really poor. Neither is the poor's physician without his remedy—he can recover his proper fees, as from a private patient; and institute legal proceedings as well.

III. Before concluding the present article, with any observations of our own upon the different systems of Rome and Prussia, for the administration of medical relief to the sick poor, we must crave the attention of onr readers to a very excellent report of the great charity infirmary at Berlin, to which allusion has already been made in the preceding pages; the Report is drawn up by RUST, and entitled "*A Summary Account of the Patients who were treated in the Charity Infirmary during the year* 1838; *and a Statement of the Alterations which have taken place in that establishment in the course of the last ten years.*

A. From this report it would appear that, at the close of the year, ending 31st Dec. 1837, there remained under treatment in the infirmary, 958 patients, of whom 893 belonged to the different parishes of Berlin, 26 to those of Potsdam; and 39 to other districts. In the year 1838, the admissions amounted to 7780, besides 341 children born in the establishment, so that the actual number of persons under medical treatment, and attended, during the year, amounted to 9079. Of these 8135 were dismissed in various ways, 6955 being cured, or having received benefit from the treatment adopted in their cases; 239 being pronounced incurable; 11 having absconded; 37 being still born; and 893 having deceased: consequently, on the 31st Dec. 1838, there remained under treatment, 944.

Of the 7750 patients, admitted during the year, 1956 were brought to the infirmary by the sanitary officers of the administration of the poor of Berlin; 401 by the police, from their different districts; 13 from the governors of the new hospital; 141 from the Orphan's Institution; 116 from the workhouse; 1 from the Penitentiary; 2 from prison; 919 from the different lock-up-houses; 5 from the goal of the district Muhlendorf; 1206 from the different manufactories (Gewerke); 437 by the surgical inspectors of the houses of ill fame; 2241 by the different medical gentlemen of the town, or, who presented themselves voluntarily for admission. Besides the above, who were all from Berlin; there were 113 admissions from Potsdam, of whom 18 were women in a state of pregnancy; and, from other districts, 229, of whom 219 were sick, and 10 pregnant.

The 9079 patients under treatment during the year, spent in the infirmary 351,905 days altogether; of which, 322,138 are to be reckoned for the 8672 Berlin patients; 8830 for those of Potsdam; and 20,937 for those from other districts. According to this statement the number of days during which each individual was under treatment, averaged about 39.

The 322,138 days illness of the 8672 Berlin patients were further divided thus:—96,851 among 2956 pay patients; and 225,285 among 5716 gratuitous patients. Of those who paid, 693 belonged to the parishes of Berlin. Of the gratuitous patients, 4893 were from Berlin; and 107 from Potsdam.

The number of the cured, and, if not cured—at least benefitted and relieved, is, in proportion to the entire number of admissions as 7·8;—those of the incurable, as 1·34; of still-born to children born alive, as 1·9.

B. In the building destined for the higher classes of pay-patients, 16 patients remained under treatment on the 31st Dec. 1837; and 105 admissions took place between that date and the 31st of December in the following year. Of these, 95 were cured, or otherwise benefitted; 4 were dismissed as incurable; 11 died, and 11 remained under treatment at the last mentioned date. The proportion of the cures to the admissions is as 6·7; of the uncured cases, as 1·30; of the deaths, as 1·11.

"It would not be uninteresting," continues one author, "particularly when taken in connexion with the above summary of results, to publish the alterations which this establishment has undergone during the last ten years :—Alterations which merit notice the more because through them the hospital acquired that form in which it has begun not only to rival all the larger establishments of a similar kind, in the neighbouring states ; but has likewise begun to exercise a governing influence upon the medical art of the whole kingdom. It is, so to speak, become the centre in which all the radii of scientific events in the practical art of healing, meet ; and, by the carefully examined results of which, by far the greater number of all the physicians in the country profit ; as, through them again, the knowledge thus acquired, is still further propagated, to the great benefit of their fellow citizens. But, in order to attain this end, it became absolutely requisite to alter and improve, according to circumstances, all the regulations of the establishment, both internal and external. Neither did former changes in the government of the infirmary, under which it throve but indifferently, seem calculated to favour rapid improvements, as, through this change, the organic concatenation of the spreading administerial maxims had been, necessarily, interrupted.

Till the year 1819 the former Poor-law Commissioners had been the governors of the establishment;—they were followed by the Royal Government; and, lastly, by the Royal Presidence of the Police. In 1828 the undersigned (Rust) was entrusted with the honourable commission to commence the long wished for re-organization of the Infirmary ; and, so far as circumstances might permit, to complete it.

In consequence of his investigations, the affairs of the state hospitals as well as the superior direction of the charity, was, in the year 1830, committed to directors, appointed by a Royal Decree, bearing date the 7th of September 1830. These directors strove to the utmost of their power to improve the administration of hospitals. They had deeply studied the science of medicine themselves, and were well acquainted with the general deficiency respecting it in Prussia; a deficiency, rendered only more evident by comparison of the progress made in other nations, and especially by the example of their infirmaries.

The extension of the buildings ; the complete re-organization of the internal regulations ; and an elevation of the moral character of the establishment, as well as the provision of adequate funds for carrying out the necessary improvements, was the difficult task they had to perform.

In order to get rid of the many evils arising from the overcrowded state of its inmates, the Directors used great exertions to get the building enlarged. The dwelling-houses of such persons as, though attached to, were not obliged to be constantly resident in, the hospital, were transformed into wards ; the admission of incurable cases, or of such persons, as had been, from destitution, accustomed to find there a temporary refuge, was entirely disallowed; and the distribution of the original and newly-acquired space was effected upon medical principles. A new hospital also, capable of holding from 3 to 400 patients, and also of future enlargement, was erected for the reception of the insane, and afterwards prepared for syphilitic and psorous cases. And by the separation of those patients a great advance was made not only to the enlarged accommodation of the whole institution, but also towards the intended object of transforming the charity into a more decent civil hospital. Separate buildings were likewise erected for patients afflicted with small-pox, cholera, or similar disorders ; and these buildings were, at the same time, fitted for the reception of independent patients, who would find in them the comfort of roomy, and, at the same time, private apartments. Another infirmary was erected in addition, adjoining yet detached from the other, and perfectly isolated. A third is nearly finished, in which, when completed, all the washing of the infirmary is to be conducted. Another building was reared for the reception of the many strangers whose necessary business leads them through the town; and also for the reception of the many unmarried people, who, in case of illness, are deprived of all the comforts of a home. *The cost of all these alterations and additional buildings amounted to two hundred and ten thousand dollars.*

Regulations were next made for ensuring stricter attention to the patients, on the part of the medical and other attendants; and perfect cleanliness in every part of the several buildings. And nothing was omitted to raise the moral character of the establishment in the eyes of the public. The patients, also, were not only separated, according to their sex and age; but likewise classified according to their respective diseases; and a directing physician appointed to every 150 patients; the number of such officers being thereby increased from 2 to 8.

Several subdivisions of the building were transformed into clinical institutions with competent masters, in which the second object of the charity, that it might become a school of practical instruction for young medical gentlemen, was achieved. The assistant medical officers, who are changed from time to time, were thus enabled to acquire practical skill in the treatment of disease themselves without risk to their poor patients, before embarking in private practice. Their operations and practice being all conducted under the special direction of the senior doctors, whose emoluments were raised according to their capacities, as were also the salaries of the nurses.

To any one who had known the institution ten years ago, the immensity as well as number of the improvements effected under the new direction, could not fail to be, at once, very perceptible. Formerly, it stood alone, and enclosed by a wall, in an unpopular and desert locality, to which, only one road led, and formed the extreme point of a thinly inhabited suburb. Now, the wall which once surrounded it has been removed several hundred yards off;—a contiguous bog which had proved pernicious to the health of the inmates, besides being a nuisance to the neighbourhood, has been transformed into a beautiful garden with which it is intended to surround all the buildings of the infirmary. The principle streets of the city leading to the 'Charity,' have been since adorned with handsome pavement, and the environs of the establishment decorated with modern and beautiful houses, though built at a certain distance from the infirmary, that all occasion of fear to the inhabitants might be removed, thereby transforming the immediate locality of the infirmary into one of the finest quarters of the city of Berlin.

The exercise of a strict but judicious economy helped very materially to remove the pecuniary difficulties with which the establishment had to contend. Among other instances, the article of diet may be mentioned. The governors, formerly, were not very careful on this point, not sufficiently considering either the recovery or the welfare of their patients. And the same was the case with medicines, bandages, &c. It is true, that such economies, in a thriving establishment, served principally to meet incidental expences in other departments, where increase was necessary and unavoidable. Nevertheless, it is proper to cite one example, on account of the considerable saving effected—it is drink, and principally that of malt liquor, of which, for many years, there had been a constant and prodigal abuse, for until the year 1828 the annual cost of this kind of drink alone reached no less a sum than 14,000 dollars, and, notwithstanding the immense augmentation of patients during the subsequent period, the consumption was so reduced, that the annual expence did not exceed from 3 to 4,000 dollars, thereby effecting a saving of from 10 to 11,000 dollars yearly."—*Rust.*

IV. If there be one principle more readily deducible than another from the facts collected in the preceding portions of this article, it is that—in order to an effectual administration of medical relief generally to the poor of any country—the management of any provision for affording such relief should be committed to a director, professionally qualified to say what is and what is not necessary; and officially confined to the single task of superintending the operations of all the subordinate instruments employed to render that relief generally applicable, and always efficient.

Where, as in England, the directing power is a Board composed of individuals, whose other duties are such as to preclude the possibility of their giving an undivided attention to the single object of affording prompt relief in time of sickness; that object can only have a share, and sometimes, perhaps, only a small share of their attention; and of necessity can only participate with other objects in a division of their sympathy, while of itself an object sufficiently vast and important to demand and to deserve undivided attention and sympathy, without which, it is obvious, it can never secure those energies or that zeal which are indispensable to its attainment.

Where, as in England, the attainment of an object, confessedly not more desirable than important, calls for an amount of professional knowledge scarcely to be looked for except in the profession itself, it follows that to entrust the control or management of any organization whereby the object in view is to be obtained to laymen, is to ask of ignorance what is consistent with knowledge alone; and to task unskilfulness for that which consummate skill alone can perform. It is the old Egyptian folly in Government—of exacting labour without ability, and compelling men to make bricks without allowing them the straw with which to make them. Such is the case in Italy. Such is the case in Germany. Such is the case in England.

In Prussia, for instance, while the hospitals are admirably managed, the medical relief of the poor at their own homes is as abominably mismanaged, where managed at all; although the history of one of the former institutions alone, since it passed into the hands of a medical director, and a consideration of the authorities by whom the other is regulated, would transform the mystery to as simple a matter.

Neither does it appear to be duly apprehended by the multitudinous ecclesiastics of modern Rome why, in their hospitals, the mortality is so frightful;—or why, in their lunatic asylum, the spectacle of men and women herded together like swine, is deemed too frightful to be exposed to the gaze of the stranger.

Priests manage the Roman hospitals. Medical relief in Prussia is provided for by municipalities. In England it is effected by a Poor-law Commission, the èleves of the ministry, and Boards of Guardians, too frequently the mere creatures of a predominant parochial faction, and indifferently composed, as in one of the parishes before our eyes of a schoolmaster, a grocer, a baker, a butcher, a mender of roads, and a mender of shoes, a keeper of the insane, and a *gentleman.*

It would surely be no more than a reasonable concession, not only to the profession, but to common sense and justice, and to the interests of the poor, were some medical knowledge infused into the "Commission." We can see no objections to the addition of a medical commissioner to the stock in esse, save perhaps the unwillingness of the latter to admit a new associate, or the lukewarmness of Government towards a class of men who cannot offer great political services. On this last score, however, we are not so sure of the policy of affronting an extensive and influential body, and the working of the New Poor-law shews, we think, the inconvenience of their opposition. When we compare the treatment of the Bar and of our own profession, we cannot but be struck with the enormous distinction which a minister makes between those who can and those who cannot brawl for the purposes of party. Is it ever to be thus?

